# Supplementary material for: Staff, resident, and care partner perceptions on the use of a personalized tablet to mitigate the impact of isolation in long-term care residents
Source: PLoS One. 2025 May 30;20(5):e0319005. doi: 10.1371/journal.pone.0319005 (PMC12124502; doi:10.1371/journal.pone.0319005)
Supplement: S1 Appendix — (DOCX) [file pone.0319005.s001.docx]

**S1 Appendix**

**Sample Interview Guide**

**Pre-DiT Interview Guide**

1. Please describe the current social distancing measures or isolation rules your care home has in place and any effects that has had on your job.

PROBES:

- 1. What are some challenges that have come with following social distancing protocols and isolation rules?
  2. How have residents responded to the changes? Can you please describe what you have observed, and what feedback you received from residents?

1. Please provide some examples of how you use technology in the LTC home.

PROBES:

a. How often is technology used?

- 1. b. How comfortable and familiar are you with using new technology? What reservations do you have?
  2. b. What have been your experiences in implementing new technology in a LTC home?

c. What do you like about the current technology that is in use? What do you dislike?

d. If it were up to you, what improvements would you make about the current technology available in your LTC home?

**Post Interview Guide:**

1. What did you enjoy about using the tablet?

PROBES:

- 1. What were some of the things about it that you liked to use?
  2. What did you not like about the tablet?
  3. What is something you were hoping the tablet could do? What suggestions do you have to improve the tablet?
  4. Describe how the tablet helped you connect with other people.

1. Can you tell me what your initial thoughts were when you heard about the opportunity for the person you care for to use the tablet?

PROBES:

- 1. What concerns did you have? Expectations? Were you looking forward to anything?
  2. How do you feel now?

1. Can you explain the ways in which the person you care for used their tablet?

PROBES:

- 1. What types of entertainment or leisure activities did they access by using the tablet?
  2. How did the person you care for use the tablet to engage in recreational activities?
  3. How did they use the tablet to communicate with others? Who did they use the tablet to communicate with and how frequently?
  4. How did their use of the tablet change over time?
