# Supplementary material for: Staff, resident, and care partner perceptions on the use of a personalized tablet to mitigate the impact of isolation in long-term care residents
Source: PLoS One. 2025 May 30;20(5):e0319005. doi: 10.1371/journal.pone.0319005 (PMC12124502; doi:10.1371/journal.pone.0319005)
Supplement: Attachment — Submitted filename: Rebuttal Letter-112124.docx [file pone.0319005.s003.docx]

RE: PONE-D-24-15124- Staff, Resident and Care Partner Perceptions on the Use of a Personalized Tablet to Mitigate the Impact of Isolation in Long-Term Care Residents

Dear Prof. Köpke,

Thank you for your interest in our manuscript and for the constructive feedback.

Below we outline the changes we have made to the manuscript, as per the feedback from the editor and reviewer. We have created an itemized list of all the reviewer comments and our responses. We have presented changes in the manuscript using ‘track’ changes

Andrea Iaboni, MD DPhil FRCPC

Geriatric Psychiatrist and Medical Lead, Seniors Mental Health, University Health Network

Senior Scientist, KITE, Toronto Rehab Research Institute

Associate Professor, Dept. of Psychiatry, University of Toronto

**Comments to the Author**

**Reviewer #1:**

1. Reviewer Comments to Authors: The paper describes experiences with a tech intervention, DIT-Tech, which aims to enhance LTCH residents’ wellbeing by addressing social isolation. The presentation of the findings is clear and interesting. However, some details about the research are missing; this information may have implications for the interpretation of the findings and/or the limitations of the research.

Response to Reviewers: Thank you for your feedback and for highlighting the clarity and interest in our presentation of DIT-Tech. To address your request for further details, we have expanded on the research design, participant selection, and the contextual factors that may influence the interpretation of findings. We have also added further discussion on the limitations of the study to ensure a more comprehensive understanding of the results and their implications.

2. Reviewer Comments to Authors: Under “Setting and intervention”, it states that 7 “diverse” LTCHs participated in the research but no details are provided. How were the homes diverse? How were LTCHs recruited?

Response to Reviewers: The term “diverse” in reference to the 7 participating LTCHs reflects differences in their geographical locations, resident populations, and available resources. We now write:

The recruitment of LTCHs involved a strategic approach, wherein potential sites were identified based on existing collaborations with the research team and their willingness to participate. The long-term care homes represented urban and rural settings and cared for residents of various socioeconomic backgrounds, and with varied resident needs. The diversity of the LTCHs was characterized in terms of representation of different regions of Ontario and size of community. Of the 7 homes, 2 were located in a large city, 3 were located in different small cities (50-150,000 people), and 2 were located in small towns (5-15,000 people). 5 were operated by not-for-profit organizations and 2 were operated by a municipality. In terms of size of the homes, they ranged between 106-228 beds per home, with an average size of 142 beds. This diversity was crucial to capture a broad spectrum of perspectives and experiences regarding the implementation of the DIT-Tech tablet intervention.

3. Reviewer Comments to Authors: No information is provided on the characteristics of residents who used the tablets. Given the high proportion of LTCH residents living with dementia, it would be particularly important to know what proportion of residents who used the tablets had dementia. This information would provide important context for interpreting the findings. If this information is not available, the potential impacts should be discussed in the limitations section. It would also be important to know if any of the residents who were interviewed had dementia and what proportion of care partners had relatives with dementia. If there is not good representation of residents with dementia and/or care partners of relatives have dementia, then the impacts of this should be discussed in the discussion and/or the limitations section. For example, future research directions are discussed on page 25 but there’s no mention of ensuring research includes the perspectives and voices of people living with dementia. Their use, and perceptions of DIT-Tech may differ from residents without dementia.

Response to Reviewers: The demographic characteristics of the full study sample are available in the publication of the primary outcomes of the DIT-Tech study, now referenced in the text.

In this paper, we have provided the demographic information of those residents and care partners involved in the interviews as part of this qualitative aim. It was an omission that we did not include information about the cognitive abilities of resident participants. Diagnosis of dementia is not accurately captured in the health administrative data available to us for this study, but all LTC residents receive a resident assessment of cognitive function. We now include the Cognitive Performance Scale (CPS) of interview participants in Table 2. Two participants did not have cognitive impairment (CPS score 0), one had mild impairment and 4 had moderate impairment.

4. Reviewer Comments to Authors: No residents were interviewed individually – all were part of interviews with care partners. Were residents active participants in the interviews? On page 9 it states that “resident quotes were largely omitted from the excerpts”, stating that there was agreement between residents’ and care partners’ comments. Why were most resident quotes (vs. care partner quotes) omitted? The tablets are intended for LTCH residents, and the aim of the study is to examine perceptions of the tablets. Given this, the perceptions of residents and staff would be the most relevant. Yet the manuscript only includes one quote from a resident. Please consider the use of additional resident quotes. Also discuss, as appropriate, the limited input/data from residents in the discussion or limitations sections.

Response to Reviewers: Thank you for your feedback regarding the engagement of residents in the interviews. We acknowledge that residents were not often engaged in the discussions and frequently echoed the sentiments expressed by their care partners, providing brief replies. We had noted “Notably, resident quotes were largely omitted from the excerpts, as they frequently echoed sentiments expressed by their care partners with brief replies. However, there were no instances of contradictory responses between dyad participants.” Furthermore, we now write:

The study primarily relied on dyad interviews with care partners and residents, which may have restricted the depth of insights from residents. Given that many residents echoed their care partners' sentiments, the findings may not fully represent the unique perspectives of residents regarding their experiences with the DIT-Tech tablets. As no individual interviews were conducted with residents, there is a missed opportunity to capture their independent thoughts and feelings about the technology, that future studies can employ.

5. Reviewer Comments to Authors: Was each tablet provided to a single resident for use? How did the personalization process work? Was it based on information provided by residents? care partners? And was this done through discussion, survey, interview or some other approach? If a tablet was provided to another resident, would it be re-programmed? Was the research team responsible for individualizing the tablet, or the staff? For what period of time / how frequently were the tablets used by residents and relatives of care partners? Also, what role did interviewed staff members play in implementing/supporting residents’ use of the tablets. This information would provide important context for interpreting the findings.

Response to Reviewers: Thank you for your comments. We now write:

Briefly, tablets were provided to a single resident for their personal use, ensuring that the technology was tailored to their individual needs and preferences. The personalization of each tablet was a collaborative effort that involved information gathered from both residents and their care partners. This process included discussions with care staff aimed at understanding the residents’ interests, preferred communication methods, and any specific applications that would enhance their engagement. If a tablet was to be reassigned to another resident, it would be reprogrammed to reflect the new resident’s preferences and requirements. The responsibility for individualizing the tablets primarily lay with the staff, who worked closely with the research team to implement the necessary customizations based on resident input and care partner feedback. The staff members interviewed played a significant role in supporting residents’ use of the tablets. They assisted with training, troubleshooting, and ongoing encouragement for residents to engage with the technology.

6. Reviewer Comments to Authors: The limitations identified in the limitations section should be expanded upon and the implication(s) of each limitation discussed.

Response to Reviewers: We have expanded our limitations. This section now reads as:

However, the study's reliance on varied technology exposure during interviews limited some participants' detailed feedback on specific features. Recruitment through LTCH site leads might introduce biases. The study primarily relied on dyad interviews with care partners and residents, which may have restricted the depth of insights from residents. Given that many residents echoed their care partners' sentiments, the findings may not fully represent the unique perspectives of residents regarding their experiences with the DIT-Tech tablets. As no individual interviews were conducted with residents, there is a missed opportunity to capture their independent thoughts and feelings about the technology, that future studies can employ. The geographic focus on Ontario also limits the study's applicability to regions with different long-term care policies, resource availability, and healthcare systems. Lastly, given the evolving nature of LTCH environments and resident needs, the study may not fully reflect the long-term sustainability or broader impact of the intervention over time.

7. Reviewer Comments to Authors: Under “Design” – there is only mention of understanding “staff” perspectives, not residents and care partners.

Response to Reviewers: We now write “This qualitative descriptive study (Sandelowski, 2010), used purposive sampling to recruit staff members, residents and care partners from multiple long-term care homes (LTCH) who were directly involved in the implementation of DIT-Tech”.

8. Reviewer Comments to Authors: Table 1 is incomplete. Information regarding ethnicity is said to be “pending”.

Response to Reviewers: Thank you for identifying this omission in Table 1This row was added incorrectly as ethnicity data was not captured for care partners. It has now been removed.

9. Reviewer Comments to Authors: Some rows in the tables include document line numbers.

Response to Reviewers: We appreciate you bringing this to our attention, and we will ensure that the tables are revised to remove any extraneous line numbers in the final version of the manuscript.

10. Reviewer Comments to Authors: DIT-Tech is sometimes written with a capital “I” and sometimes with a lower case “I”.

Response to Reviewers: Thank you for pointing out the inconsistency in the capitalization of "DIT-Tech." We will ensure that the term is uniformly presented with a capital “I” throughout the manuscript to maintain clarity and consistency. Your feedback is greatly appreciated.

11. Reviewer Comments to Authors: Line 454 – should this say “…differing from shared technology studies”?

Response to Reviewers: Thank you for your observation regarding Line 454. It should indeed read “differing from shared technology studies.”

12. Reviewer Comments to Authors: Line 469 – there is a reference to “leisure wellbeing” but elsewhere well-being (hyphenated) was usually described as mental well-being.

Response to Reviewers: Thank you for pointing out the inconsistency regarding the term "leisure wellbeing." We have revised Line 469 to ensure that it aligns with the hyphenated "mental well-being" used elsewhere in the manuscript.

**Reviewer #2**

13. Reviewer Comments to Authors: Thank you for the opportunity to review this manuscript which aimed to develop an understanding of the staff, resident and care partner experiences, including barriers and facilitators, to the adoption of remote-access personal tablets in long-term care home.

Response to Reviewers: Thank you for your thoughtful review of our manuscript. We appreciate your recognition of the study's aim to understand the experiences of staff, residents, and care partners regarding the adoption of remote-access personal tablets in long-term care homes.

14. Reviewer Comments to Authors: Abstract: Please explain the abbreviation LTCH.

Response to Reviewers: We now write “This study aimed to develop an understanding of the staff, resident and care partner experiences, including barriers and facilitators, to the adoption of remote-access personal tablets in long-term care home (LTCH).”

15. Reviewer Comments to Authors: Setting and intervention: The description of the intervention and the setting is not very detailed. Please describe some typical characteristics of long-term care homes for an international readership (e.g. ownership, size related to resident places, implemented care concepts, qualification levels of staff). The intervention is a complex intervention. Please use the recommendations of the TiDier Guideline to describe the intervention. For example, it is currently unclear who used the tablets, when, how often and under what circumstances. It is also unclear what role and qualification the DIT Tech Coordinator had?
    [https://www.equator-network.org/reporting-guidelines/tidier/#:~:text=The%20TIDieR%20Checklist%20is%20available%20to%20download%20as%20a%20PDF](https://urldefense.com/v3/__https:/www.equator-network.org/reporting-guidelines/tidier/*:*:text=The*20TIDieR*20Checklist*20is*20available*20to*20download*20as*20a*20PDF__;I34lJSUlJSUlJSU!!CjcC7IQ!KlX3RNgE2He5lOeJ8FQJAxF6ZSfjJfwnfPBZAok_EKik1Y3MIIqVsFsPJGGjzRi2MSVAqggKdTFe-TR__A$)

Response to Reviewers: Thank you for your comments. We have added these additional details as well as referencing a publication which provides further details about the methods and results of the primary aim of this study.

We now write:

The recruitment of LTCHs involved a strategic approach, wherein potential sites were identified based on existing collaborations with the research team and their willingness to participate. The long-term care homes represented urban and rural settings and cared for residents of various socioeconomic backgrounds, and with varied resident needs. The diversity of the LTCHs was characterized in terms of representation of different regions of Ontario and size of community. Of the 7 homes, 2 were located in a large city, 3 were located in different small cities (50-150,000 people), and 2 were located in small towns (5-15,000 people). 5 were operated by not-for-profit organizations and 2 were operated by a municipality. In terms of size of the homes, they ranged between 106-228 beds per home, with an average size of 142 beds. This diversity was crucial to capture a broad spectrum of perspectives and experiences regarding the implementation of the DIT-Tech tablet intervention. These Android tablets, property of the LTCHs, feature standard apps and Skype. Key features include remote control access, direct family calls via auto-answer on Skype, and personalized setups for residents (Astell et al., 2024).

Full study details on the implementation of the tablet are available in the larger study’s manuscript (Astell et al., 2024).

Briefly, tablets were provided to a single resident for their personal use, ensuring that the technology was tailored to their individual needs and preferences. The personalization of each tablet was a collaborative effort that involved information gathered from both residents and their care partners. This process included discussions with care staff aimed at understanding the residents’ interests, preferred communication methods, and any specific applications that would enhance their engagement. If a tablet was to be reassigned to another resident, it would be reprogrammed to reflect the new resident’s preferences and requirements. The responsibility for individualizing the tablets primarily lay with the staff, who worked closely with the research team to implement the necessary customizations based on resident input and care partner feedback. The staff members interviewed played a significant role in supporting residents’ use of the tablets. They assisted with training, troubleshooting, and ongoing encouragement for residents to engage with the technology.

In our study, "tablet usage level" refers to the frequency and extent of interaction residents had with the tablets, which can include the duration of use, types of applications accessed, and engagement in activities facilitated by the tablets. The median tablet use by participants was 7 minutes per day.

16. Reviewer Comments to Authors: Participants and Recruitment: What does tablet usage level mean? What was the distribution of the tablet usage level.? Please present the tablet usage level as a result in terms of the degree of implementation of your intervention.

Response to Reviewers: Thank you for your inquiry regarding the term "tablet usage level." We now write

“*In our study, "tablet usage level" refers to the frequency and extent of interaction residents had with the tablets, which can include the duration of use, types of applications accessed, and engagement in activities facilitated by the tablets. The median tablet use by participants was 7 minutes per day*.”

17. Reviewer Comments to Authors: Resident participant characteristics: What were the reasons for the isolation of the residents? To what extent did the duration of isolation have an influence on the selection of residents?

Response to Reviewers: Thank you for your questions regarding the characteristics of resident participants and the reasons for their isolation. The residents in our study experienced isolation primarily due to public health measures during the COVID-19 pandemic. This is made explicit in the introduction where we define isolation (line 57), and in the description of the goals of the tablet. Being isolated for infection control and prevention reasons was not a requirement for participation in the study or the interviews. We have edited the participant recruitment section in the methods to be more explicit about how residents were included in the interviews.

18. Staff participant characteristics: Please describe the qualifications and roles of the employees so that an international readership can categorise them (e.g. using an international qualification framework)

Response to Reviewers: Thank you for your request regarding the qualifications and roles of employees in long-term care homes. We have added this information in a footnote to Table 3..

19. Response to Reviewers: Discussion: Please reflect on future scientific steps necessary for the development and evaluation of the analysed intervention? In particular, please reflect on the necessity of investigating the effectiveness in corresponding study designs.

Response to Reviewers: Thank you for your feedback. We now write

shared technology in facilities[28].

The primary outcomes of this intervention were published elsewhere (Astell et al., 2024), revealed that there was a large variability in tablet use across participants, with a median of 7 minutes per day (with an interquartile range of 27) throughout the study (Astell et al., 2024). Higher tablet usage was linked to factors such as younger age, better cognitive function, no hearing impairments, and having a care partner living farther away. However, there were no measurable improvements in quality of life, recreational activities, or relational closeness based on quantitative assessments. In contrast, this study’s qualitative insights highlighted that motivation among staff and care partners was a critical facilitator of tablet use. The ability of DIT-Tech to offer personalized experiences tailored to residents’ preferences was perceived as enhancing cognitive stimulation, social engagement, and quality of life. Additionally, the qualitative emphasis on perceived, short-term benefits—such as moments of engagement or autonomy—points to a possible mismatch between the outcomes valued by participants and the metrics used in the quantitative evaluation. However, resistance emerged as a substantial barrier, driven by staff concerns about increased workloads, technological literacy gaps, and skepticism regarding older adults’ capacity or willingness to use tablets. The study highlights a contrast between the initial enthusiasm and subsequent challenges encountered during implementation. Motivation among staff and family members was underpinned by the potential of DIT-Tech to supplement caregiving and recreation efforts, with personalized features supporting meaningful engagement. The relationship between motivation and effective implementation underscores the importance of prioritizing and harnessing stakeholder motivation when implementing technology in LTCH. However, implementation does not equate to adoption as technology can be offered or rolled out but not adopted, (i.e., incorporated into an individual’s daily life) [29]. However, resistance, fueled by past negative experiences with technology and limited resources for training and troubleshooting, dampened the broader adoption of the technology. Unlike residents and family members who valued personalization, many staff viewed the process of customizing devices as burdensome, particularly in the context of existing staffing shortages. These findings underscore the importance of addressing structural barriers, such as providing adequate training and technological support, to reduce resistance and enhance adoption.

[…]

Research could examine the long-term sustainability of remote-access technology, including the financial and logistical considerations necessary to maintain these systems over time. Finally, future studies might assess the role of training and support for staff and care partners to ensure that the full potential of these technologies is realized, particularly in terms of ease of use and optimal engagement with residents. By following LTCHs over extended periods, researchers can also identify trends in social isolation, mental health outcomes, and the evolving integration of technology into care practices.

Astell, A., Dosanjh, S., D'Elia, T., Kokorelias, K. M., Stewart, S., Grigorovich, A., . . . Iaboni, A. (2024). Personalized Tablets for Residents in Long-Term Care to Support Recreation and Mitigate Isolation. *Journal of the American Medical Directors Association, 25*(7), 105022.

Sandelowski, M. (2010). What's in a name? Qualitative description revisited. *Res Nurs Health, 33*(1), 77-84.
